# Oxidative Stress and Mitochondrial Dysfunction in Chronic Kidney Disease: From Molecular Mechanisms to Biomarkers and Targeted Therapies

**DOI:** 10.3390/antiox15091116

**Published:** 2026-09-04

**Authors:** Federica De Luca, Dario Troise, Valentina Camporeale, Giorgia Leccese, Federica Galloso, Roberto Cuttano, Barbara Infante, Giovanni Stallone, Elena Ranieri, Giuseppe Stefano Netti

**Affiliations:** 1 Unit of Clinical Pathology, Department of Medical and Surgical Sciences, University of Foggia, University Hospital “Policlinico Riuniti”, Viale Luigi Pinto, 71122 Foggia, Italy; federica.deluca@unifg.it (F.D.L.); valentina.camporeale@unifg.it (V.C.); giorgia_leccese.555553@unifg.it (G.L.); federica_galloso.563213@unifg.it (F.G.); roberto.cuttano@unifg.it (R.C.); elena.ranieri@unifg.it (E.R.); 2 Center for Research and Innovation in Medicine (CREATE), Department of Medical and Surgical Sciences, University of Foggia, University Hospital “Policlinico Riuniti”, Viale Luigi Pinto, 71122 Foggia, Italy; dario.troise@unifg.it (D.T.); barbara.infante@unifg.it (B.I.); giovanni.stallone@unifg.it (G.S.); 3 Unit of Nephrology, Dialysis and Transplantation, Advanced Research Center on Kidney Aging (A.R.K.A.), Department of Medical and Surgical Sciences, University of Foggia, University Hospital “Policlinico Riuniti”, Viale Luigi Pinto, 71122 Foggia, Italy; 4 Cancer Cell Signalling Unit, Fondazione IRCCS “Casa Sollievo della Sofferenza”, 71013 San Giovanni Rotondo, Italy

**Keywords:** chronic kidney disease (CKD), mitochondrial dysfunction, mitophagy (PINK1/Parkin), NADPH oxidase 4 (NOX4), reactive oxygen species (ROS), mitochondria-targeted therapy

## Abstract

Chronic kidney disease (CKD) represents a major global health challenge, affecting more than 10% of the population and contributing substantially to morbidity and premature mortality. Growing evidence identifies oxidative stress and mitochondrial dysfunction as central drivers of renal injury and disease progression across diverse etiologies. The kidney is one of the most mitochondria-rich organs in the body, reflecting the high bioenergetic demands required for tubular reabsorption and metabolic homeostasis. Disruption of mitochondrial oxidative phosphorylation, excessive production of reactive oxygen species (ROS), and impaired mitochondrial quality control mechanisms promote tubular injury, inflammation, and fibrosis. In particular, dysfunction of the electron transport chain, activation of NADPH oxidase isoforms—especially NOX4—and alterations in mitochondrial dynamics create a vicious cycle of oxidative damage and bioenergetic failure. Emerging evidence highlights the importance of mitochondrial quality control pathways, including fusion–fission balance, PINK1/Parkin-mediated mitophagy, and mitochondrial biogenesis regulated by PGC-1α and TFAM. Additional mechanisms include ferroptosis, epigenetic regulation, mitochondrial DNA-mediated innate immune activation, and Na^+^/K^+^-ATPase-linked redox signaling. At the translational level, redox and mitochondrial biomarkers and targeted therapies are biologically compelling, but the evidence is uneven: most candidate biomarkers remain insufficiently standardized, and direct mitochondria-targeted interventions are supported predominantly by preclinical studies or small human proof-of-concept trials. This review therefore emphasizes not only mechanistic advances but also conflicting findings, model limitations, and the barriers that currently separate experimental efficacy from clinically meaningful CKD outcomes.

## 1. Introduction

The kidney is among the most metabolically active organs in the human body, characterized by high oxygen consumption and an exceptionally dense mitochondrial network that supports its complex physiological functions. In fact, the renal cortex—particularly the proximal tubular epithelium—contains one of the highest mitochondrial densities among mammalian tissues, surpassed only by the myocardium [1]. This remarkable abundance reflects the enormous bioenergetic requirements associated with maintaining renal homeostasis, including glomerular filtration, electrolyte balance, acid–base regulation, and active reabsorption of solutes and water. Approximately 180 L of glomerular filtrate is processed by the kidneys daily, and the majority of this filtrate is reabsorbed in the proximal tubules through energy-dependent transport mechanisms that rely heavily on mitochondrial adenosine triphosphate (ATP) production [1,2,3].

Mitochondria generate ATP primarily through oxidative phosphorylation (OXPHOS), a process driven by the mitochondrial electron transport chain (ETC) located within the inner mitochondrial membrane [1,4,5]. Electrons derived from metabolic substrates are transferred through ETC complexes I–IV, ultimately reducing oxygen to water while generating a proton gradient that powers ATP synthesis. While this system is highly efficient, it also represents a major source of reactive oxygen species (ROS), which are generated as unavoidable byproducts of electron leakage from ETC complexes—particularly complexes I and III [1,6]. Under physiological conditions, low levels of ROS function as signaling molecules that regulate cellular processes such as proliferation, differentiation, and stress responses. However, excessive ROS production or impaired antioxidant defenses disrupt cellular redox homeostasis, leading to oxidative stress and damage to lipids, proteins, and nucleic acids [7,8,9].

Recent advances in molecular nephrology have revealed that mitochondrial dysfunction is not only a consequence of renal injury but also a central driver of disease initiation and progression. Emerging pathogenic mechanisms—including ferroptosis, epigenetic regulation of mitochondrial genes, and metabolic reprogramming—have further expanded our understanding of how oxidative stress and mitochondrial alterations contribute to renal pathology [4,10,11,12,13]. Moreover, novel therapeutic strategies targeting mitochondrial pathways are currently being investigated, raising the possibility that restoring mitochondrial homeostasis may represent a promising approach for preventing or slowing CKD progression [4,10,14,15].

This review critically examines the molecular mechanisms linking oxidative stress and mitochondrial dysfunction to chronic kidney disease. We focus on the major renal sources of reactive oxygen species, mitochondrial quality-control pathways, disease-specific redox phenotypes, redox and mitochondrial biomarkers, and established or emerging therapeutic strategies. Animal and cellular studies provide the mechanistic core of the review, whereas human observations and early intervention studies are retained selectively to evaluate whether experimental mechanisms have translated beyond model systems. Particular attention is given to study limitations, conflicting findings, and the distinction between mechanistic plausibility, translational signals, and clinically validated renal benefit.

### Literature Search Strategy and Evidence Profile

This narrative review was developed through iterative searches of PubMed/MEDLINE, Scopus, and Web of Science for literature published from January 2000 through 31 July 2026, supplemented by backward citation tracking and targeted retrieval of seminal earlier studies. Search concepts combined “chronic kidney disease” or “CKD” with terms including “oxidative stress”, “reactive oxygen species”, “mitochondrial dysfunction”, “mitophagy”, “mitochondrial dynamics”, “ferroptosis”, “NOX4”, “Na/K-ATPase”, “cardiotonic steroids”, “mineralocorticoid receptor”, “obesity”, “MASLD/MASH”, “GLP-1 receptor agonist”, “biomarker”, and “therapy”. Because this is a narrative rather than a systematic review, searches were iterative and no PRISMA-style deduplicated initial retrieval count was generated; to avoid a retrospective and potentially misleading record count, we report the final included bibliography and its evidence profile. The final bibliography comprises 216 references. Primary studies were categorized by their dominant evidence source; mixed-design studies were assigned to the highest translational level represented (human > animal > in vitro). Reviews, guidelines, and methodological articles were tabulated separately (Table 1). Human evidence was not excluded a priori because it was used specifically to assess translational maturity and discordance with experimental findings, rather than to provide a comprehensive clinical guideline-level synthesis.

## 2. Primary Sources of Oxidative Stress in the Renal Parenchyma

**Figure 1:**
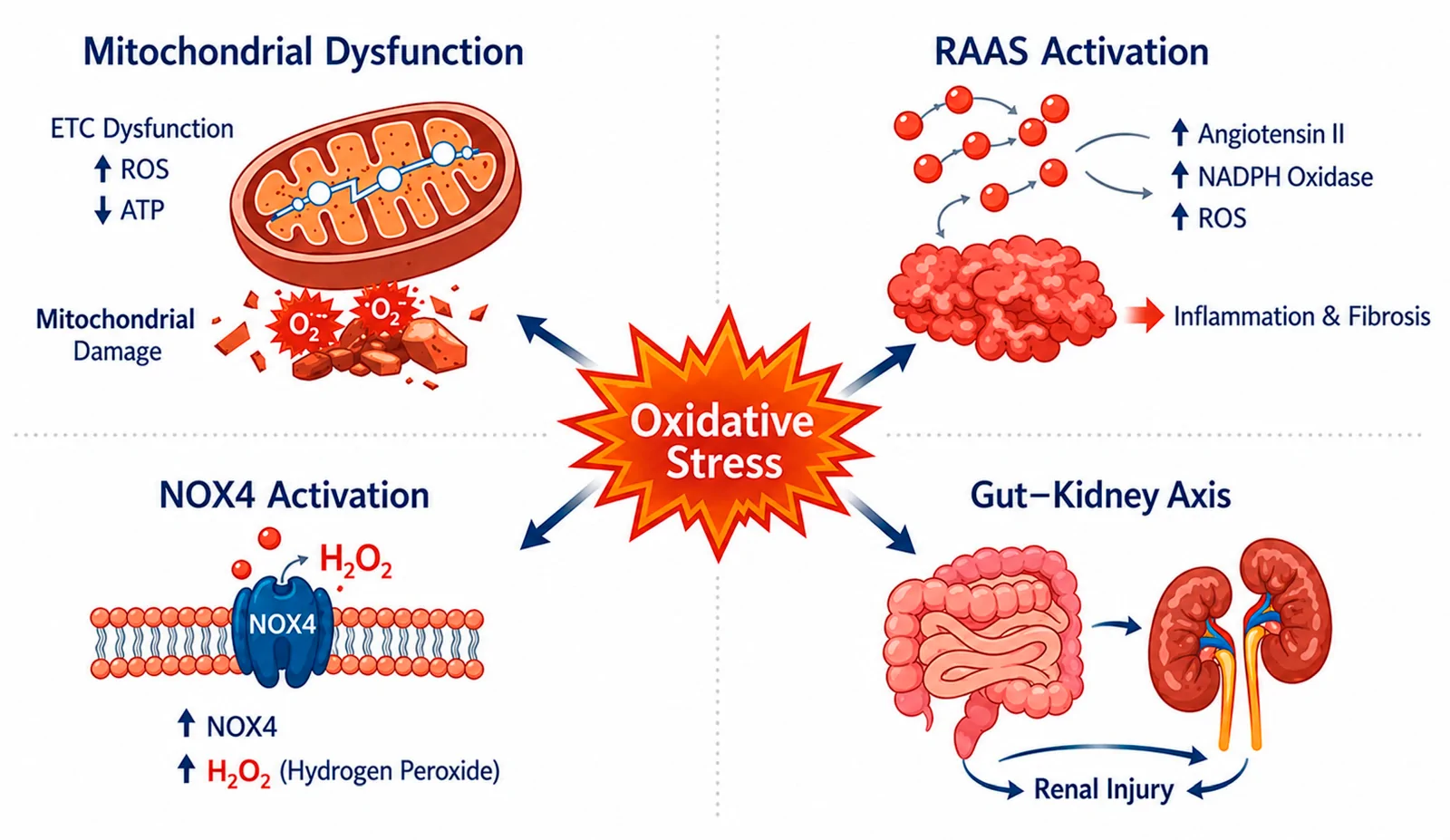
Sources of oxidative stress in chronic kidney disease. Major contributors to oxidative stress in CKD include mitochondrial electron transport chain dysfunction, activation of NADPH oxidases—particularly NOX4—renin–angiotensin–aldosterone system (RAAS) activation, and gut–kidney axis dysregulation with accumulation of uremic toxins. These pathways converge to amplify reactive oxygen species generation, inflammation, and renal injury. Created in BioRender. Colangelo, T. (2026) https://BioRender.com/lxbldl0.

Oxidative stress represents a fundamental pathogenic mechanism in chronic kidney disease and reflects an imbalance between reactive oxygen species (ROS) generation and cellular antioxidant capacity. In the kidney, ROS arise from interconnected mitochondrial and non-mitochondrial sources, including electron transport chain dysfunction, NADPH oxidases, chronic renin–angiotensin–aldosterone system (RAAS) activation, metabolic substrate excess in diabetes, and the accumulation of uremic toxins associated with gut dysbiosis. Hyperglycemia is particularly relevant because excess substrate flux, advanced glycation end products (AGEs), lipotoxicity, and impaired AMPK/SIRT1 signaling converge on mitochondrial ROS generation and NOX4 activation. These processes reinforce inflammation, cellular injury, and progressive renal fibrosis (Figure 1) [7,8,10,16,17,18,19,20,21,22,23,24].

### 2.1. Mitochondrial Electron Transport Chain Dysfunction and Superoxide Leakage

Mitochondria represent the primary endogenous source of reactive oxygen species in most mammalian cells. During oxidative phosphorylation (OXPHOS), electrons derived from metabolic substrates are transferred through the mitochondrial electron transport chain, which consists of five multiprotein complexes embedded within the inner mitochondrial membrane. Although the ETC is designed to efficiently transfer electrons to molecular oxygen, a small proportion of electrons prematurely escape the chain and react with oxygen to form the superoxide radical (O_2_•^−^) [1,25,26].

Under physiological conditions, mitochondrial ROS production is tightly controlled by antioxidant defense systems such as superoxide dismutases (SODs), catalase, glutathione peroxidase, and peroxiredoxins [14,27,28,29]. In CKD, hyperglycemia, lipid overload, hypoxia, inflammation, and uremic stress can increase reducing-equivalent delivery to the ETC, impair respiratory complex function, and promote electron leakage. Complexes I and III are major sites of superoxide generation. Structural and functional ETC abnormalities have been documented across complementary CKD models, including streptozotocin-induced and db/db diabetic models, 5/6 nephrectomy, and unilateral ureteral obstruction (UUO), although no single model reproduces the full heterogeneity of human CKD [8,11,25,26,30,31].

In proximal tubular epithelial cells, mitochondrial dysfunction is particularly detrimental due to their heavy reliance on fatty acid oxidation (FAO) as a primary energy source. Disruption of FAO leads to the accumulation of lipid intermediates, including long-chain acylcarnitines, which interfere with mitochondrial metabolism and further increase ROS production. This process contributes to lipotoxicity, mitochondrial fragmentation, and the progressive decline of cellular bioenergetic capacity [32]. In addition, oxidative damage to mitochondrial DNA can impair the synthesis of essential ETC proteins encoded by the mitochondrial genome, further compromising mitochondrial respiration and establishing a self-perpetuating cycle of oxidative stress and mitochondrial dysfunction [30,33].

Mitochondrial injury is also relevant when CKD develops after an episode of acute kidney injury. Rather than representing a separate focus of this review, AKI is considered here specifically as an initiating event for the AKI-to-CKD transition: persistent mitochondrial ROS production, incomplete recovery of oxidative phosphorylation, and maladaptive inflammatory signaling can impair tubular repair and favor chronic fibrosis after severe ischemic or nephrotoxic injury [33,34,35,36,37,38,39,40].

### 2.2. NADPH Oxidase Signaling: The Central Role of NOX4

While mitochondria generate ROS as byproducts of metabolism, the NADPH oxidase (NOX) family of enzymes produces ROS as its primary biological function. Among the seven NOX isoforms identified in mammals, NOX4 is the predominant isoform expressed in renal tissue and is particularly abundant in tubular epithelial cells, podocytes, and endothelial cells [41,42,43,44,45].

Unlike other NOX isoforms that primarily generate superoxide, NOX4 predominantly produces hydrogen peroxide (H_2_O_2_), a relatively stable ROS that can diffuse across cellular compartments and participate in redox signaling. Under physiological conditions, NOX4-derived ROS contribute to cellular signaling pathways involved in differentiation and metabolic adaptation. However, chronic activation of NOX4 has been implicated in the pathogenesis of multiple kidney diseases, including diabetic kidney disease, hypertensive nephropathy, and renal fibrosis [42,43,44,45,46,47,48].

In diabetic kidney disease, hyperglycemia and advanced glycation end products stimulate NOX4 expression in podocytes and mesangial cells, leading to increased ROS production and activation of pro-fibrotic signaling pathways such as transforming growth factor-β (TGF-β) signaling. Increased NOX4 activity has also been linked to podocyte apoptosis, glomerulosclerosis, and albuminuria in experimental models of diabetic nephropathy [18,19,20,21,43,49].

Recent studies have identified the SH3YL1 protein as an important cytosolic activator that interacts with NOX4 to enhance ROS generation in renal cells [4]. Disruption of the SH3YL1–NOX4 interaction has been shown to reduce oxidative stress and attenuate renal fibrosis in experimental models, highlighting this signaling axis as a potential therapeutic target. Furthermore, several pharmacological compounds—including polyphenols and AMPK activators—have been shown to suppress NOX4 expression and mitigate oxidative stress in diabetic kidney disease [16,22,23,24].

### 2.3. RAAS Activation and Redox Crosstalk

The renin–angiotensin–aldosterone system (RAAS) plays a central role in regulating blood pressure, fluid balance, and renal hemodynamics. However, chronic activation of RAAS is also a major contributor to oxidative stress and inflammation in CKD. Angiotensin II (Ang II), the principal effector peptide of RAAS, exerts multiple deleterious effects on renal tissue through activation of the angiotensin II type 1 receptor (AT_1_R) [41,45,50].

Binding of Ang II to AT_1_R stimulates the activation of NADPH oxidases, particularly NOX4, leading to increased ROS production and oxidative damage in renal cells. This ROS generation initiates a cascade of signaling events that activate transcription factors such as nuclear factor-κB (NF-κB) and activator protein-1 (AP-1), promoting the expression of pro-inflammatory cytokines, chemokines, and adhesion molecules. As a result, immune cells—including macrophages and T lymphocytes—are recruited to the kidney, further amplifying inflammatory responses and promoting tissue injury [41,45,50].

Moreover, Ang II-induced ROS production can impair mitochondrial function by damaging mitochondrial DNA, disrupting mitochondrial membrane potential, and promoting mitochondrial fragmentation. This interaction between RAAS signaling and mitochondrial dysfunction establishes a feedback loop in which oxidative stress perpetuates inflammation and fibrosis, ultimately contributing to progressive renal damage [8,30,41,45,51,52,53].

### 2.4. Na^+^/K^+^-ATPase Signaling, Cardiotonic Steroids, and Electrolyte–Redox Crosstalk

The Na+/K+-ATPase is indispensable for renal tubular transport because its ATP-dependent pumping function maintains transmembrane Na+ and K+ gradients that drive secondary solute reabsorption. Beyond this canonical transport function, pioneering studies by Xie, Shapiro, and colleagues established that Na+/K+-ATPase can also act as a signal transducer. In cardiac myocytes, Xie et al. first demonstrated that ouabain-initiated Na+/K+-ATPase signaling rapidly generates intracellular ROS that function as second messengers in downstream growth-related pathways. Subsequent work showed that the Na+/K+-ATPase α1 subunit and Src form a functional signaling complex whose activity is regulated by cardiotonic steroid binding. More specifically, Yan et al. demonstrated that ROS-dependent carbonylation of the Na+/K+-ATPase α1 subunit participates in a feed-forward Na+/K+-ATPase/c-Src/ROS signaling mechanism in renal proximal tubular cells, linking redox signaling to sodium transport. These foundational observations provide the mechanistic basis for the Na+/K+-ATPase/Src/ROS amplification loop. In UUO-associated renal fibrosis, pharmacologic interruption of Src or NADPH oxidases reduced oxidative markers and fibrotic remodeling, and complementary LLC-PK1 experiments implicated NOX2/NOX4 in this loop [54,55,56,57]. Thus, Na+/K+-ATPase can be viewed not only as an energy-consuming pump vulnerable to mitochondrial ATP depletion, but also as a redox-sensitive signaling node capable of linking tubular transport stress to inflammation and fibrosis.

Endogenous cardiotonic steroids (CTSs; also termed digitalis-like factors), including marinobufagenin (MBG) and endogenous ouabain, have been proposed as endogenous ligands of Na+/K+-ATPase. An early study by Kennedy et al. from the Xie/Shapiro group provided important causal experimental evidence linking elevated MBG to the pathophysiology of renal failure. In partially nephrectomized rats, circulating MBG increased substantially; chronic MBG administration reproduced systemic oxidant stress, cardiac hypertrophy, fibrosis, and impaired diastolic relaxation, whereas immunization against MBG attenuated major components of this phenotype. These findings supported the concept that increased endogenous CTS concentrations in renal failure are not merely epiphenomena but may actively participate in Na+/K+-ATPase-dependent pathological signaling. Sodium/volume expansion and impaired renal clearance may favor accumulation of these factors, while extracellular K+ availability directly influences pump activity and also intersects with aldosterone/MR signaling. In a small human CKD study, circulating MBG concentration was higher and erythrocyte Na+/K+-ATPase activity lower than in controls, with parallel findings in partially nephrectomized rats [58,59]. These observations raise the possibility that MBG or related factors could serve as biomarkers or therapeutic targets. However, the field remains controversial: reported circulating concentrations are generally very low, assay specificity varies, and the concentrations required for canonical pump inhibition or signaling differ across Na+/K+-ATPase isoforms and experimental systems [60]. Accordingly, neither endogenous cardiotonic steroids nor serum sodium/potassium should currently be considered specific biomarkers of renal mitochondrial dysfunction; their translational value requires standardized assays and prospective validation.

### 2.5. The Gut–Kidney Axis and Uremic Toxins

In addition to intrinsic renal sources of oxidative stress, increasing attention has been directed toward the role of the gut–kidney axis in the pathogenesis of chronic kidney disease. Alterations in intestinal microbiota composition and metabolism—collectively referred to as dysbiosis—contribute to the generation and systemic accumulation of uremic toxins that exacerbate oxidative stress and inflammation in CKD [17,61,62,63,64].

In patients with impaired renal function, the reduced clearance of microbial metabolites leads to the accumulation of several protein-bound uremic toxins, among which indoxyl sulfate (IS) and p-cresyl sulfate (pCS) are the most extensively studied. These metabolites originate from the bacterial metabolism of dietary amino acids such as tryptophan and tyrosine and undergo hepatic conjugation before entering systemic circulation. Because of their strong binding affinity to albumin, these toxins are poorly removed by conventional dialysis and progressively accumulate as kidney function declines [62,63,65,66,67].

Indoxyl sulfate has been identified as a potent inducer of oxidative stress in renal tubular epithelial cells and vascular endothelial cells. Mechanistically, IS stimulates ROS production through the activation of NADPH oxidases, particularly NOX4, and enhances mitochondrial oxidative stress. Moreover, IS interferes with endogenous antioxidant defense systems by suppressing the activity of nuclear factor erythroid 2-related factor 2 (Nrf2), a transcription factor that regulates the expression of numerous antioxidant and detoxification enzymes [65,68]. The inhibition of Nrf2 signaling results in decreased expression of protective proteins such as heme oxygenase-1 (HO-1), glutathione peroxidase, and superoxide dismutase, thereby exacerbating oxidative damage within renal tissue [27,28,68,69,70].

Beyond its direct pro-oxidant effects, indoxyl sulfate also promotes inflammatory signaling pathways that contribute to renal fibrosis. Activation of the aryl hydrocarbon receptor (AhR) by IS triggers downstream signaling cascades that stimulate the production of pro-inflammatory cytokines and profibrotic mediators, including transforming growth factor-β (TGF-β) and connective tissue growth factor (CTGF). These processes accelerate tubulointerstitial fibrosis, which represents a key determinant of CKD progression [61,65,71].

The systemic consequences of gut-derived uremic toxins extend beyond the kidney. Elevated circulating levels of IS and pCS have been strongly associated with endothelial dysfunction, vascular calcification, and increased cardiovascular risk in CKD patients. Oxidative stress induced by these toxins contributes to mitochondrial damage in vascular cells, thereby linking renal dysfunction with the heightened cardiovascular morbidity observed in CKD populations [50,61,65,72,73].

Importantly, therapeutic strategies aimed at modulating the gut microbiota—including dietary interventions, probiotics, prebiotics, and oral adsorbents—are currently being investigated as potential approaches to reduce uremic toxin burden and mitigate oxidative stress in CKD. Although clinical evidence remains limited, these strategies highlight the growing recognition of the gut–kidney axis as an important contributor to oxidative stress and mitochondrial dysfunction in chronic kidney disease [61,63,71,74].

## 3. Mitochondrial Quality Control (MQC) Failure

Mitochondrial homeostasis is maintained by a highly coordinated network of processes collectively referred to as mitochondrial quality control (MQC). These mechanisms ensure the preservation of mitochondrial integrity and functionality through the dynamic regulation of mitochondrial morphology, selective removal of damaged organelles, and synthesis of new mitochondria. The MQC system consists primarily of three interconnected processes: mitochondrial dynamics (fusion and fission), mitophagy, and mitochondrial biogenesis [6,75,76].

In the healthy kidney, these processes work in concert to maintain an efficient mitochondrial network capable of meeting the high metabolic demands of renal tubular cells. However, in chronic kidney disease, multiple stressors—including oxidative stress, metabolic dysregulation, and inflammatory signaling—disrupt MQC pathways, leading to the accumulation of dysfunctional mitochondria. These damaged organelles generate excessive reactive oxygen species, impair ATP production, and activate apoptotic and inflammatory pathways that contribute to progressive renal injury [4,8,30,51,75,77].

**Figure 2:**
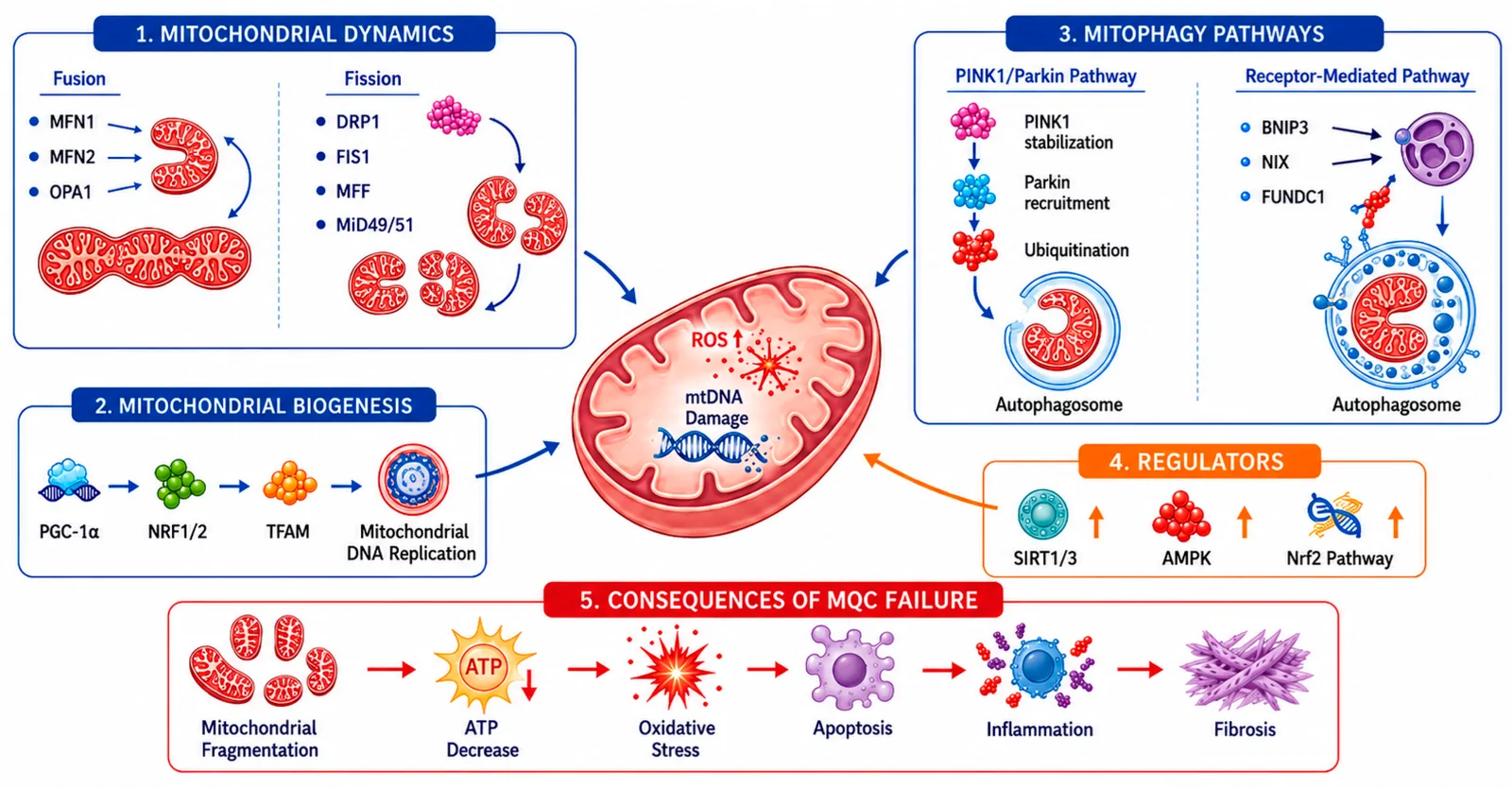
Mitochondrial quality control pathways in chronic kidney disease. Mitochondrial quality control (MQC) is maintained through the coordinated regulation of mitochondrial dynamics, biogenesis, and mitophagy. (1) Mitochondrial dynamics include fusion, mainly regulated by mitofusin 1 (MFN1), mitofusin 2 (MFN2), and optic atrophy 1 (OPA1), and fission, primarily mediated by dynamin-related protein 1 (DRP1), fission 1 (FIS1), mitochondrial fission factor (MFF), and MiD49/51. (2) Mitochondrial biogenesis is controlled by the PGC-1α/NRF1/2/TFAM axis, which promotes mitochondrial DNA replication and renewal of the mitochondrial network. (3) Mitophagy pathways remove damaged mitochondria through both the canonical PINK1/Parkin pathway, which involves PINK1 stabilization, Parkin recruitment, ubiquitination, and autophagosome formation, and receptor-mediated mitophagy, driven by BNIP3, NIX, and FUNDC1. (4) Key regulators, including SIRT1/3, AMPK, and the Nrf2 pathway, support adaptive mitochondrial homeostasis and antioxidant responses. When these MQC mechanisms fail, damaged mitochondria accumulate, leading to increased ROS production, mtDNA damage, and impaired energy metabolism. (5) The downstream consequences of MQC failure include mitochondrial fragmentation, ATP depletion, oxidative stress, apoptosis, inflammation, and fibrosis, ultimately contributing to CKD progression. Created in BioRender. Colangelo, T. (2026) https://BioRender.com/lxbldl0.

MQC dysfunction may also determine whether an acute mitochondrial insult re-solves or evolves into chronic disease. Experimental AKI models show early fragmentation and impaired mitophagy, but their relevance to a CKD-focused review lies in persistent mitochondrial damage after incomplete recovery: sustained ATP deficiency, ROS generation, and maladaptive repair can promote the AKI-to-CKD transition through chronic inflammation and fibrosis (Figure 2) [6,34,36,37,39].

### 3.1. Mitochondrial Dynamics: The Balance Between Fusion and Fission

Mitochondrial dynamics balance fusion, mediated mainly by MFN1/2 and OPA1, with DRP1-dependent fission. Fusion supports exchange of mitochondrial contents and network integrity, whereas fission segregates damaged components for removal. In CKD, this balance commonly shifts toward fragmentation, with increased activating DRP1 phosphorylation and reduced fusion proteins reported across experimental renal injury models [78,79,80,81,82,83,84].

Excessive fission is not merely a morphological feature: fragmented mitochondria have lower respiratory efficiency, generate more ROS, and facilitate cytochrome-c release and profibrotic signaling. Nevertheless, DRP1 activity is context-dependent, and complete suppression of fission could also impair physiological mitochondrial turnover; the therapeutic objective is restoration of dynamic balance rather than indiscriminate fission blockade [8,51,78,85,86].

### 3.2. Mitophagy Pathways: The PINK1/Parkin Cascade

Mitophagy selectively removes dysfunctional mitochondria through the autophagy-lysosome system and limits persistence of ROS-generating organelles [6,76,87,88,89,90]. In the canonical PINK1/Parkin pathway, loss of mitochondrial membrane potential stabilizes PINK1 on the outer membrane, activates Parkin-dependent ubiquitination, and recruits LC3-linked autophagy receptors for lysosomal clearance [88,91,92].

Evidence from acute injury models is mechanistically informative for the AKI-to-CKD transition. Genetic loss of PINK1 or Parkin worsens mitochondrial ROS accumulation and inflammatory signaling after renal injury, whereas preserved mitophagy favors organelle clearance and adaptive repair [11,34,38,87,93]. These observations support a protective role for mitophagy, but most data remain preclinical and cannot be directly extrapolated to chronic human CKD.

In addition to the PINK1/Parkin pathway, receptor-mediated mitophagy pathways have also been described. Proteins such as BNIP3, NIX, and FUNDC1 contain LC3-interacting regions (LIRs) that allow them to directly recruit autophagosomes without requiring ubiquitination. These pathways are particularly important during hypoxic stress and have been implicated in renal ischemia and diabetic nephropathy [6,76,87,89,90].

When mitochondrial clearance remains insufficient during maladaptive repair, defective mitophagy may contribute to CKD progression. Human evidence is still limited and is largely associative; altered expression of mitophagy-related pathways has been linked to reduced kidney function and fibrosis, but standardized longitudinal measures of mitophagic flux in patients are not available [11,51,75,87].

### 3.3. Mitochondrial Biogenesis and the PGC-1α Signaling Axis

Mitochondrial biogenesis replenishes the organelle pool and is coordinated primarily by PGC-1α, which activates NRF1/2 and TFAM-dependent programs controlling respiratory proteins and mtDNA replication [94,95,96,97]. PGC-1α signaling is frequently reduced in CKD, contributing to lower mitochondrial mass, impaired fatty-acid oxidation, and diminished ATP-generating capacity [8,11,51,94].

AMPK and NAD+-dependent sirtuins, particularly SIRT1/SIRT3, provide upstream metabolic control of PGC-1α and antioxidant responses. Their dysfunction links metabolic stress and aging to impaired mitochondrial renewal. Thus, CKD-associated MQC failure reflects the combined loss of appropriate dynamics, selective clearance, and replacement rather than an isolated defect in one pathway [16,94,98,99,100,101].

From a translational perspective, MQC pathways are mechanistically compelling but the evidence base remains uneven. Much of the causal evidence derives from genetically modified rodents, UUO, toxin-induced injury, ischemia–reperfusion, or cultured tubular/podocyte systems, each of which captures only selected components of chronic human disease. Measurements of mitochondrial morphology, membrane potential, ROS, and autophagic flux are also method-dependent. Therefore, apparently concordant pathway changes across models should not be interpreted as proof that manipulating a single MQC node will improve hard renal outcomes in patients. Human biopsy, longitudinal biomarker, and intervention studies remain the principal gap.

## 4. Emerging Pathogenic Axes in Renal Pathology

Beyond classical oxidative stress pathways, recent advances in molecular nephrology have uncovered several additional mechanisms that connect mitochondrial dysfunction to renal injury. Among these, ferroptosis, epigenetic regulation of mitochondrial genes, and non-coding RNA signaling have emerged as critical contributors to oxidative stress amplification and disease progression in chronic kidney disease (CKD). These mechanisms integrate metabolic disturbances, mitochondrial dysfunction, and inflammatory signaling, forming complex pathogenic networks that drive tubular injury and fibrosis [6,30].

### 4.1. Ferroptosis: Iron-Dependent Lipid Peroxidation

**Figure 3:**
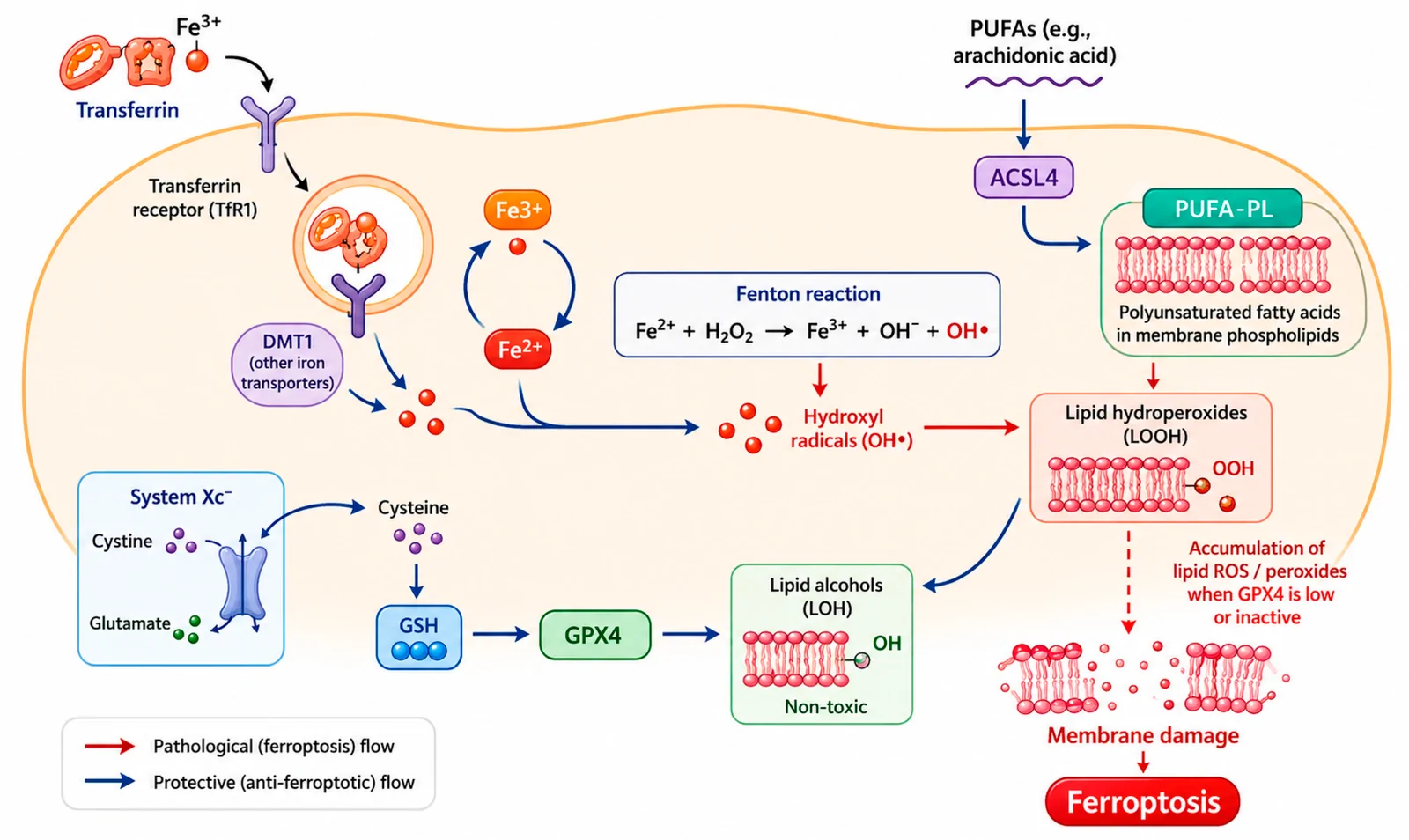
Molecular mechanisms of ferroptosis in renal cells. Ferroptosis is an iron-dependent form of regulated cell death driven by uncontrolled lipid peroxidation. Transferrin-mediated iron uptake and intracellular Fe^2+^/Fe^3+^ redox cycling promote the Fenton reaction, generating hydroxyl radicals that initiate peroxidation of polyunsaturated fatty acids incorporated into membrane phospholipids (PUFA-PL). ACSL4 facilitates PUFA incorporation into membrane phospholipids, thereby increasing susceptibility to lipid hydroperoxide formation. The major anti-ferroptotic defense is the System Xc^−^/cystine/GSH/GPX4 axis, in which GPX4 uses glutathione to reduce toxic lipid hydroperoxides to non-toxic lipid alcohols. When GPX4 activity is reduced or lost, lipid ROS and lipid peroxides accumulate, leading to membrane damage and ferroptotic cell death. Created in BioRender. Colangelo, T. (2026) https://BioRender.com/lxbldl0.

Ferroptosis is an iron-dependent regulated cell-death program driven by unchecked phospholipid peroxidation. Redox-active Fe^2+^ promotes hydroxyl-radical formation, while ACSL4 enriches membranes in oxidation-prone polyunsaturated phospholipids. The principal protective axis is System Xc^−^/glutathione/GPX4, which detoxifies lipid hydroperoxides; loss of GPX4 permits membrane damage and ferroptotic death (Figure 3) [102,103,104,105,106,107,108,109,110,111,112,113,114,115,116].

In CKD-relevant settings, ferroptosis has been implicated in diabetic kidney disease and in maladaptive repair after ischemic or nephrotoxic AKI that can progress to CKD. Pharmacological inhibitors such as ferrostatin-1 or liproxstatin-1 attenuate renal injury in experimental systems, supporting the proof-of-concept for ferroptosis as a target; however, these findings remain predominantly preclinical and do not yet establish efficacy or safety in chronic human kidney disease [35,102,103,106,112].

Another important regulatory pathway controlling ferroptosis is the nuclear factor erythroid 2-related factor 2 (Nrf2) signaling pathway. Nrf2 is a transcription factor that regulates the expression of numerous antioxidant and cytoprotective genes, including those involved in glutathione synthesis and iron metabolism. Activation of Nrf2 enhances cellular resistance to ferroptosis by increasing GPX4 expression and restoring redox balance. Conversely, impaired Nrf2 signaling—frequently observed in CKD—facilitates lipid peroxidation and ferroptotic cell death [6,27,28,68,69,70,116].

The close relationship between ferroptosis and mitochondrial dysfunction further highlights the central role of oxidative stress in renal pathology. Mitochondrial ROS production can amplify lipid peroxidation, while mitochondrial iron accumulation can increase susceptibility to ferroptotic injury. These interactions establish ferroptosis as a key mediator linking mitochondrial dysfunction with tubular cell death and progressive renal damage [6,30,104,105,107,108].

### 4.2. Epigenetic Regulation of Mitochondrial Function

Epigenetic regulation provides a mechanism by which metabolic and oxidative stress can produce persistent changes in mitochondrial programs. DNA methylation and histone modifications influence genes involved in oxidative metabolism, antioxidant defense, inflammation, and mitochondrial biogenesis [12,13,117,118,119]. Altered methylation of mitochondrial-related loci, including PMPCB and TFAM, has been associated with kidney disease and may impair protein processing or mtDNA maintenance.

Histone acetylation and methylation similarly couple cellular metabolism to mitochondrial phenotype. Changes in H3K27ac and EZH2-dependent H3K27me3 can alter fission-, fibrosis-, and antioxidant-related transcription, while metabolites such as S-adenosylmethionine provide substrates for chromatin-modifying enzymes [12,13,117,118,119]. These findings support a metabolic-epigenetic feedback loop, but most causal data remain experimental and kidney-cell specificity is incompletely resolved.

### 4.3. Non-Coding RNAs and Mitochondrial Regulation

Non-coding RNAs, particularly microRNAs (miRNAs), represent another important layer of regulation linking oxidative stress and mitochondrial dysfunction in kidney disease. MicroRNAs are short RNA molecules that regulate gene expression by binding to complementary sequences in target mRNAs, leading to translational repression or mRNA degradation [120,121,122].

Several microRNAs have been identified as regulators of mitochondrial dynamics and metabolism in renal cells. For instance, miR-668 has been shown to inhibit mitochondrial fission by targeting mitochondrial protein 18 (MTP18), thereby protecting renal tubular cells from apoptosis during ischemic injury. By suppressing excessive mitochondrial fragmentation, miR-668 helps preserve mitochondrial function and cellular viability [121,122].

Conversely, miR-17 has been implicated in the suppression of peroxisome proliferator-activated receptor alpha (PPAR-α), a transcription factor involved in fatty acid oxidation and mitochondrial metabolism. Downregulation of PPAR-α by miR-17 impairs mitochondrial energy metabolism and promotes cyst formation in polycystic kidney disease models [94,120,121,122].

These regulatory networks demonstrate how epigenetic and post-transcriptional mechanisms converge to influence mitochondrial function and oxidative stress responses. Understanding these pathways may provide new opportunities for therapeutic intervention aimed at restoring mitochondrial homeostasis in CKD [120,121,122,123,124].

## 5. Organelle Crosstalk: Mitochondria–Endoplasmic Reticulum Interactions

Mitochondria do not function as isolated organelles but instead participate in complex interactions with other cellular compartments. Among these interactions, communication between mitochondria and the endoplasmic reticulum (ER) plays a particularly important role in maintaining cellular homeostasis [79,83].

These interactions occur at specialized contact sites known as mitochondria-associated membranes (MAMs). MAMs facilitate the transfer of lipids, calcium ions, and metabolic intermediates between the ER and mitochondria, thereby coordinating cellular metabolism, calcium signaling, and stress responses [79,83,84].

In renal cells, MAMs are essential for regulating mitochondrial calcium uptake. Calcium ions released from the ER are transferred directly to mitochondria through channels such as the inositol 1,4,5-trisphosphate receptor (IP3R), voltage-dependent anion channel (VDAC), and mitochondrial calcium uniporter (MCU). Controlled calcium transfer stimulates mitochondrial metabolism by activating dehydrogenases involved in the tricarboxylic acid (TCA) cycle [4,79,83].

However, excessive calcium transfer from the ER to mitochondria can lead to mitochondrial calcium overload, which disrupts mitochondrial membrane potential and triggers opening of the mitochondrial permeability transition pore (mPTP). This event results in mitochondrial swelling, release of pro-apoptotic factors, and activation of cell death pathways [30,79,125].

Disruption of MAM signaling has been implicated in various forms of kidney disease. Oxidative stress and mitochondrial dysfunction can alter ER–mitochondria communication, leading to abnormal calcium signaling and increased susceptibility to apoptosis [30,79,125].

Another important consequence of mitochondrial dysfunction is the release of mitochondrial DNA into the cytoplasm. Because mtDNA resembles bacterial DNA in structure, it can be recognized by innate immune receptors as a danger signal. One of the most important pathways activated by cytosolic mtDNA is the cyclic GMP–AMP synthase–stimulator of interferon genes (cGAS–STING) pathway [125,126,127,128,129].

Activation of cGAS by mtDNA leads to the production of cyclic GMP–AMP (cGAMP), which activates STING on the ER membrane. STING signaling subsequently induces the production of type I interferons and pro-inflammatory cytokines, promoting inflammatory responses within the kidney [126,127].

Persistent activation of the cGAS–STING pathway has been implicated in chronic kidney disease and renal fibrosis. In experimental models, inhibition of STING signaling reduces inflammation and attenuates kidney injury, highlighting the importance of mitochondrial–immune crosstalk in renal pathology [126,127].

## 6. Disease-Specific Redox Mechanisms

Although oxidative stress and mitochondrial dysfunction represent common pathogenic mechanisms across different kidney diseases, the molecular pathways involved vary according to the underlying etiology, the dominant injured renal compartment, and the nature of the initiating insult [30,51]. In some settings, redox imbalance arises primarily from metabolic overload and mitochondrial substrate excess; in others, it is driven by podocyte injury, complement activation, chronic inflammation, or genetically determined alterations in calcium handling and cellular bioenergetics. Consequently, oxidative stress should not be regarded as a uniform downstream event, but rather as a disease-modifying process that acquires distinct molecular features in different nephropathies.

### 6.1. Diabetic Kidney Disease (DKD)

Diabetic kidney disease is the leading cause of chronic kidney disease worldwide and accounts for a substantial proportion of end-stage renal disease cases [16,130,131,132]. In this setting, hyperglycemia promotes a persistent state of metabolic oversupply that enhances mitochondrial electron flux, increases superoxide production, and favors oxidative injury to mitochondrial DNA, respiratory proteins, and membrane lipids [16,18,19,20,22,130,133,134,135,136,137]. However, the redox phenotype of DKD cannot be explained by mitochondrial ROS generation alone. Advanced glycation, lipotoxic stress, intrarenal RAAS activation, and inflammatory cytokines converge on ROS-generating systems, particularly NOX4, thereby amplifying oxidative injury and linking metabolic stress to structural renal damage.

In diabetic kidneys, excessive glucose exposure suppresses the activity of AMP-activated protein kinase (AMPK), a key metabolic sensor that normally supports mitochondrial biogenesis, fatty acid oxidation, and antioxidant defense [16,18,22,23,133,135,136,137]. Reduced AMPK signaling favors NOX4 upregulation and thereby intensifies ROS production in podocytes and tubular cells [16,22,23,24,49]. This interaction is mechanistically relevant because NOX4-derived oxidant signaling contributes not only to cellular injury but also to activation of profibrotic pathways, including transforming growth factor-β signaling, extracellular matrix deposition, and albuminuria progression. Experimental observations indicating that metformin restores AMPK activity and attenuates oxidative stress further support the view that mitochondrial dysfunction in DKD reflects defective adaptation to metabolic overload rather than a passive consequence of hyperglycemia alone [16,22,23,24,49].

Another important feature of DKD is the close integration between redox balance and mitochondrial quality control. Hyperglycemia suppresses the SIRT1–FOXO1 signaling axis, which normally promotes antioxidant gene expression, autophagy, and resistance to apoptosis [16,99,100,134,135]. Loss of this protective pathway renders renal cells more vulnerable to oxidative injury, especially in podocytes and proximal tubular epithelial cells, where sustained mitochondrial stress rapidly translates into cytoskeletal instability, slit diaphragm dysfunction, and fibrogenic signaling. Thus, the disease-specific redox signature of DKD is best understood as the combined result of mitochondrial ROS overproduction, impaired AMPK-dependent metabolic adaptation, NOX4 amplification, and defective SIRT1-mediated stress resistance.

### 6.2. Focal Segmental Glomerulosclerosis (FSGS)

Focal segmental glomerulosclerosis is characterized by podocyte injury/loss and seg-mental scarring. Because podocytes have limited regenerative capacity, ATP deficiency, altered mitochondrial dynamics, and ROS accumulation can destabilize the actin cyto-skeleton and amplify detachment from the glomerular basement membrane [138,139,140,141,142,143]. Both mitochondrial ROS and NOX activity contribute to this redox burden, while Nrf2-dependent defenses appear protective.

Experimental studies implicate Nrf2/Keap1, HIF-1α-ANGPTL4, and miRNA-linked pathways in the transition from podocyte stress to sclerosis; several antioxidant compounds improve experimental injury [138,139,144,145]. However, these interventions have not established clinical efficacy in primary FSGS, illustrating the translational gap between mechanistic models and a heterogeneous human disease.

### 6.3. Autosomal Dominant Polycystic Kidney Disease (ADPKD)

In ADPKD, PKD1/PKD2 dysfunction couples abnormal calcium signaling with metabolic reprogramming, reduced oxidative phosphorylation, mitochondrial stress, and proliferative cyst growth [146,147,148]. Oxidative abnormalities can precede advanced structural disease, and Nrf2/GPX4 activation attenuates lipid peroxidation and cystogenesis in experimental models [149,150].

AMPK activation provides a second metabolic checkpoint by restraining mTOR-dependent growth and chloride-driven cyst fluid secretion. Metformin, PF-06409577, and steviol reduce cystic expansion experimentally, although the relative roles of CFTR and other conductances such as TMEM16A may vary across models [151,152,153,154,155]. Thus, ADPKD illustrates redox-metabolic coupling rather than a uniform ROS-driven phenotype.

### 6.4. Membranous Nephropathy (MN)

Membranous nephropathy is an autoimmune glomerular disease and a major cause of adult nephrotic syndrome, characterized by subepithelial immune complex deposition and diffuse thickening of the glomerular basement membrane [156,157,158]. In this setting, oxidative stress is closely linked to complement-mediated podocyte injury rather than to metabolic substrate excess or primary mitochondrial disease. Immune complex deposition at the podocyte surface perturbs intracellular signaling, promotes inflammatory activation, and contributes to mitochondrial dysfunction, thereby amplifying structural damage to the filtration barrier. Experimental data increasingly support the concept that redox injury is an active component of the effector phase of MN rather than a nonspecific by-product of glomerular injury [159,160,161,162]. Recent primary evidence has further shown that complement stimulation can induce podocyte pyroptosis in MN and that mitochondrial dysfunction with ROS generation is a key component of this process.

The antioxidant response appears to be relevant in both experimental and potentially translational terms. Activation of the Nrf2/HO-1 pathway has been associated with attenuation of renal injury in experimental MN, and agents such as Sanqi oral solution, crocin, and curcumin have been shown to reduce oxidative stress and improve podocyte injury through Nrf2-centered pathways [159,160,161,162]. In parallel, autophagy-related mechanisms intersect with redox regulation. Salvianolic acid B and the combination of metformin with rapamycin have been reported to enhance autophagic responses and improve podocyte injury, supporting the view that mitochondrial quality control and oxidative stress are mechanistically linked in MN [163,164]. Ferroptotic mechanisms may also participate in disease progression. Passive Heymann nephritis studies have shown altered GPX4, GSH, and ACSL4 expression together with renal iron deposition, suggesting that ferritinophagy-associated ferroptosis may represent an additional oxidative injury pathway in MN [165,166,167,168]. Thus, in MN, oxidative stress appears to function as a bridge between immune complex/complement injury, mitochondrial dysfunction, and downstream podocyte loss.

### 6.5. Lupus Nephritis (LN)

Lupus nephritis combines immune-complex/complement activation with oxidative and mitochondrial injury across podocytes, mesangial cells, infiltrating leukocytes, and the systemic inflammatory milieu [169,170,171,172,173,174,175,176,177]. Biopsy and experimental data support excessive DRP1-mediated fission, including C5a-C5aR1-linked mitochondrial fragmentation in podocytes, as a potential bridge between complement activation and structural glomerular injury [175,176].

Nrf2, SIRT1, ROS/TRPM2/Ca^2+^, and NLRP3 pathways provide mechanistic links between redox imbalance and inflammation; citral, epigallocatenin-3-gallate (EGCG), and N-acetylcysteine have shown experimental or limited clinical signals [169,170,171,172,173,174,175,176,177,178,179,180,181,182]. The evidence nonetheless remains insufficient to use oxidative-stress markers to guide routine LN therapy, and immunologic disease control remains the clinically validated priority.

## 7. Translational Evaluation: Redox and Mitochondrial Biomarkers in CKD

Conventional CKD markers such as serum creatinine, estimated glomerular filtration rate (eGFR), and albuminuria quantify functional or structural consequences of disease but do not directly report redox state or mitochondrial injury. Experimental studies have therefore stimulated interest in F2-isoprostanes, oxidized nucleosides, protein-oxidation products, and mitochondrial DNA (mtDNA). Human studies provide useful translational signals, but most are associative and do not establish that a biomarker is kidney-specific, causal, or capable of improving clinical decisions beyond established CKD measures [15,73,183].

For this reason, the biomarkers summarized below are presented according to an evidence hierarchy rather than as clinically established tests. Preclinical studies support their mechanistic relationship to oxidative or mitochondrial injury, whereas available human cohorts mainly address detectability and association with disease phenotypes. The key unresolved questions are analytical standardization, renal specificity, temporal stability, and prospective incremental value over eGFR and albuminuria (Table 2) [15,73,183,184].

### 7.1. Lipid Peroxidation Markers

Lipid peroxidation is a hallmark of oxidative stress and occurs when reactive oxygen species attack polyunsaturated fatty acids within cellular membranes. Among the most reliable biomarkers of lipid peroxidation are F2-isoprostanes, which are prostaglandin-like compounds generated through free radical-mediated oxidation of arachidonic acid [185,186]. F2-isoprostanes are chemically stable and relatively specific indicators of in vivo oxidative stress; elevated plasma and urinary concentrations have been reported in CKD [2,15,73,183,185,186]. However, the literature is dominated by relatively small observational cohorts and heterogeneous analytical workflows. Mass-spectrometry-based methods provide the greatest specificity, whereas immunoassays are more accessible but may be affected by cross-reactivity, making universal thresholds difficult to define.

### 7.2. DNA Oxidation Markers

Oxidative damage to DNA represents another important consequence of redox imbalance in CKD. One of the most widely studied biomarkers of DNA oxidation is 8-hydroxy-2′-deoxyguanosine (8-OHdG), which results from ROS-induced modification of guanine residues within DNA. 8-OHdG reflects oxidative DNA injury and has been associated with adverse renal and cardiovascular phenotypes in CKD. These observations are biologically plausible but not kidney-specific: smoking, inflammation, diabetes, age, and other comorbidities influence systemic oxidative DNA damage, while substantial between-assay variability limits comparison across cohorts [2]. Accordingly, 8-OHdG is better regarded as a research marker of oxidative burden than as a validated predictor for individual CKD management.

### 7.3. Protein Oxidation Markers

Advanced oxidation protein products (AOPPs) represent another class of oxidative stress biomarkers relevant to CKD. AOPPs are formed through oxidative modification of plasma proteins, particularly albumin, by chlorinated oxidants generated during inflammatory processes [50,66,67,183,187]. Elevated circulating levels of AOPPs have been associated with endothelial dysfunction and CKD progression, but these relationships are vulnerable to confounding by systemic inflammation, albumin concentration and oxidative modification, dialysis-related factors, and comorbidity [50,66,67,183,187]. Their limited specificity therefore restricts interpretation as a direct readout of renal mitochondrial injury.

### 7.4. Mitochondrial Biomarkers

Beyond traditional oxidative stress markers, increasing attention has been directed toward biomarkers reflecting mitochondrial damage. Among these, circulating mitochondrial DNA (mtDNA) has emerged as a promising indicator of mitochondrial dysfunction and can also act as Damage-Associated Molecular Patterns (DAMPs) [126,128,184]. Its mechanistic link to cellular stress is strong, but the translational literature remains heterogeneous. Associations with kidney injury severity are sensitive to sample processing, cellular contamination, DNA extraction, normalization strategy, and extra-renal sources of circulating mtDNA [126,127,128,184,188]. Urinary mtDNA may be more kidney-proximal, yet reference intervals and clinically actionable cut-offs have not been standardized.

Importantly, none of the redox or mitochondrial markers summarized in Table 2 is currently validated as a routine surrogate end point for CKD progression. Most human studies are cross-sectional, single-center, or modest in size, and positive associations do not demonstrate that changing the biomarker changes the disease course. Pre-analytical handling, normalization, analytical platform, systemic inflammation, smoking, diabetes, cardiovascular disease, medication use, and reduced renal clearance can all influence concentrations. Prospective multicenter validation and demonstration of incremental value beyond eGFR and albuminuria are therefore required before these measurements can move from mechanistic research to clinical decision-making.

### 7.5. Critical Interpretation of Biomarker Evidence

The limited clinical evidence is nevertheless informative because it defines the boundary of current translation. The discrepancy between robust experimental effects and weaker clinical discrimination identifies where the field needs better study design: longitudinal sampling, kidney-specific normalization, harmonized reference materials, prespecified thresholds, and evaluation of whether biomarkers predict or mediate treatment response. Thus, human observations are retained here as a concise test of translational maturity, not as evidence that these biomarkers are ready for routine use.

## 8. Therapeutic Strategies: From Preclinical Rationale to Clinical Translation

**Figure 4:**
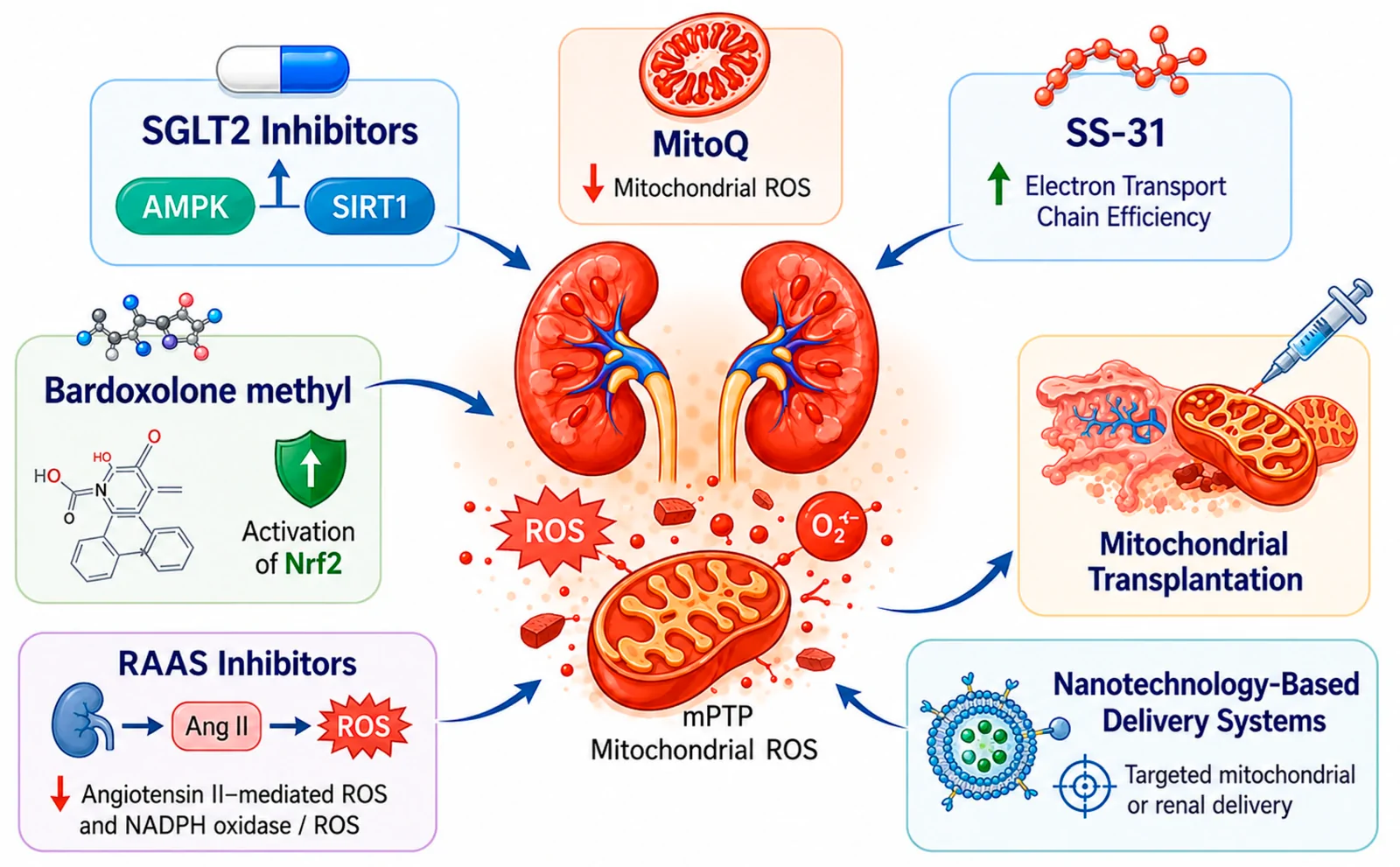
Selected therapeutic approaches targeting mitochondrial dysfunction in chronic kidney disease. The figure illustrates an evidence continuum rather than equivalent clinical readiness. SGLT2 inhibitors and RAAS-targeted therapies have established nephroprotective effects, although their mitochondrial actions are largely inferred from experimental studies. Direct mitochondria-targeted agents such as MitoQ and SS-31 (elamipretide), as well as mitochondrial transplantation and targeted nanodelivery, remain supported predominantly by preclinical or early proof-of-concept evidence in renal disease. This distinction is essential when interpreting mechanistic promise versus demonstrated clinical benefit. Created in BioRender. Colangelo, T. (2026) https://BioRender.com/lxbldl0.

Therapeutic manipulation of redox and mitochondrial pathways spans a wide evidence continuum. Some established nephroprotective therapies have reproducible kidney outcome benefits but only indirect or incompletely proven mitochondrial mechanisms, whereas direct mitochondria-targeted agents show strong experimental effects with much more limited human renal evidence. We therefore distinguish mechanistic efficacy in animal and cellular models from clinical efficacy in CKD and use the available human studies primarily to assess translational maturity rather than to provide a comprehensive treatment review (Figure 4, Table 3) [8,14,16,189,190].

### 8.1. Established CKD Therapies with Redox- and Mitochondria-Relevant Effects

Several therapies with established nephroprotective roles also intersect with redox and mitochondrial pathways, but this mechanistic overlap should not be equated with proof that mitochondrial modulation mediates their clinical benefit [8,14,16,189,190].

Sodium–glucose cotransporter-2 (SGLT2) inhibitors reduce CKD progression across multiple clinical settings [191,192,193,194]. Experimental studies support AMPK/SIRT1 activation, reduced mTOR signaling, improved autophagy, altered substrate utilization, and lower oxidative stress as potential contributors [16,22,23,24]. However, these effects coexist with major hemodynamic and metabolic actions; human outcome trials do not isolate mitochondrial mechanisms, and the extent to which mitochondrial remodeling contributes to renal protection remains uncertain.

Renin–angiotensin–aldosterone system (RAAS) inhibitors reduce Ang II-dependent NADPH oxidase activation and fibrosis-associated redox signaling in experimental models [41,45,50,195]. Their clinical renoprotection is well established, but again the antioxidant component is mechanistically entangled with changes in intraglomerular pressure, blood pressure, inflammation, and extracellular-matrix signaling rather than representing a direct mitochondria-targeted effect.

Mineralocorticoid receptor antagonists (MRAs) provide a further example of this distinction. Aldosterone/MR signaling can stimulate NADPH oxidases, mitochondrial ROS generation, NLRP3 activation, and profibrotic transcription; experimental tubular studies show attenuation of these pathways with MR blockade [196]. Clinical studies with spironolactone and finerenone support renal and cardiovascular benefit in selected CKD populations [197,198,199,200], but they do not establish that redox or mitochondrial effects are the dominant mediators. Hyperkalemia and changes in renal function also constrain use, underscoring the gap between a mechanistically attractive pathway and intervention-level risk-benefit in patients.

GLP-1 receptor agonists (GLP-1RAs) are relevant to the metabolic-redox interface of CKD because weight loss, improved glycemic control, natriuresis, reduced lipotoxicity, and lower inflammatory burden can secondarily improve mitochondrial substrate handling. Semaglutide has demonstrated kidney outcome benefit in type 2 diabetes with CKD [201], while MASH data support activity across the broader metabolic disease axis [202]. Nevertheless, these clinical outcomes do not prove a direct renal mitochondrial mechanism; the mitochondrial component remains largely inferred from experimental studies and should be interpreted as one element of a pleiotropic therapeutic effect.

### 8.2. Mitochondria-Targeted Antioxidants

Conventional antioxidant strategies have frequently failed to reproduce the magnitude of benefit predicted by experimental models, motivating the development of compounds designed to reach mitochondrial compartments more selectively [8,14,189,190]. Even with targeted delivery, however, translation remains difficult because mitochondrial exposure, disease stage, redox compartmentalization, and the balance between pathological ROS excess and physiological redox signaling differ substantially between experimental systems and patients.

MitoQ, a mitochondria-targeted ubiquinone derivative, improves oxidative stress, mitophagy-related abnormalities, and tubular injury in several experimental kidney models, including diabetic kidney disease [203]. Human renal evidence is far more limited. In a randomized, placebo-controlled pilot study of 18 adults with stage 3–4 CKD, four weeks of MitoQ improved brachial artery flow-mediated dilation but did not improve exercise capacity and was not designed to test CKD progression or structural renal outcomes [204]. Thus, the study provides a short-term vascular proof-of-concept signal rather than evidence of disease-modifying kidney efficacy.

SS-31 (elamipretide) binds cardiolipin and can improve electron transport-chain efficiency, mitochondrial respiration, and ROS handling in experimental renal injury [189,190,205,206]. A phase 2a proof-of-concept study in 14 patients undergoing stent revascularization for atherosclerotic renal artery stenosis (6 elamipretide, 8 placebo) reported attenuation of post-procedural hypoxia and favorable renal blood-flow and kidney function signals [207]. However, the sample was very small, the intervention was peri-procedural, and renovascular ischemia represents a highly selected context; these results cannot be generalized to progressive, etiologically heterogeneous CKD.

Taken together, MitoQ and elamipretide illustrate both the promise and the current limitations of direct mitochondrial therapy: biological target engagement can be demonstrated in models and small translational studies, yet evidence for durable reduction in CKD progression remains insufficient.

### 8.3. Activation of the Nrf2 Antioxidant Pathway

The Nrf2 signaling pathway represents one of the most important cellular defense mechanisms against oxidative stress. Activation of Nrf2 promotes the transcription of numerous antioxidant and cytoprotective genes, including heme oxygenase-1 (HO-1), glutathione synthesis enzymes, and detoxification proteins [27,28,68,69,70,116].

Bardoxolone methyl, a potent Nrf2 activator, provides an instructive example of the difficulty of translating redox-pathway activation. Early clinical studies in diabetic CKD showed increases in eGFR [208,209,210], but an eGFR signal is not necessarily synonymous with structural kidney protection and may reflect hemodynamic or systemic effects in addition to antioxidant biology.

The BEACON trial was terminated early because of excess heart-failure events in the bardoxolone group, whereas subsequent studies using different selection strategies renewed interest in the pathway [68,209,210,211,212]. The conflicting clinical experience emphasizes that systemic activation of an antioxidant transcriptional program can have context-dependent physiological consequences and that pathway efficacy in cells or animals does not guarantee an acceptable human risk–benefit profile.

### 8.4. Emerging Therapeutic Approaches

In addition to pharmacological interventions, several innovative strategies are being explored to restore mitochondrial function in kidney disease [189,190].

Mitochondrial transplantation represents a novel approach involving the delivery of functional mitochondria into injured tissues. Experimental studies have shown that transplantation of healthy mitochondria can restore cellular bioenergetics and reduce inflammation in models of kidney injury [189,190].

Nanotechnology-based drug delivery systems also offer promising opportunities for targeted therapy. Nanoparticles designed to accumulate in renal mitochondria can deliver antioxidants or therapeutic molecules directly to sites of oxidative damage while minimizing systemic side effects [189,190].

These approaches remain at an early experimental stage. Before clinical testing can meaningfully address efficacy, key challenges include reproducible mitochondrial delivery, tissue and cell-type specificity, persistence of the transferred or delivered cargo, immunologic and off-target effects, scalable manufacturing, and standardized pharmaco-dynamic measures of renal mitochondrial target engagement.

The therapeutic evidence should therefore be interpreted hierarchically. SGLT2 inhibitors, RAAS blockade, MR antagonism, and GLP-1RAs have clinical outcome evidence in defined CKD populations, but their proposed antioxidant and mitochondrial mechanisms are largely inferred from experimental or translational studies. Conversely, direct mitochondrial interventions show clearer mechanistic target specificity but far less clinical renal evidence. This asymmetry is informative because it highlights the difference between mechanistic target specificity and demonstrated clinical effectiveness.

### 8.5. Translational Gaps and Conflicting Evidence

Several factors plausibly explain why mitochondria-targeted antioxidants and related interventions often show larger effects in preclinical studies than in human CKD. Experimental models usually reproduce one dominant injury pathway, use genetically and environmentally homogeneous animals, and frequently administer therapy before or near the onset of injury. Patients with CKD are generally older, have more comorbidities and etiologically heterogeneous disease, receive multiple background therapies, and are often studied after fibrosis and nephron loss are established. Dose scaling and systemic pharmacokinetics do not guarantee adequate exposure in the relevant renal cell population or mitochondrial compartment, and few trials directly quantify renal target engagement.

A second issue is biological and methodological. ROS are not uniformly harmful: low-level, compartment-specific oxidant signals participate in adaptive signaling, so indiscriminate ROS suppression is biologically distinct from selectively correcting pathological mitochondrial ROS excess. Preclinical endpoints such as tissue ROS, membrane potential, ATP content, histologic injury, or short-term albuminuria may also fail to predict sustained eGFR decline or kidney failure. Small samples, short follow-up, surrogate endpoints, model-specific effect sizes, limited representation of sex and age diversity, and publication bias further increase the risk of overestimating translatability. Future studies should therefore pair clinically meaningful renal outcomes with validated pharmacodynamic biomarkers, demonstrate kidney and mitochondrial target engagement, and test interventions in aged and comorbid models that better approximate human CKD.

## 9. Lifestyle, Metabolism, and Hormonal Influences

Lifestyle factors and metabolic conditions play important roles in modulating oxidative stress and mitochondrial function in the kidney [213].

Obesity is an increasingly important driver of CKD through both indirect pathways (type 2 diabetes, hypertension, sleep apnea, and cardiovascular disease) and direct renal mechanisms, including glomerular hyperfiltration, adipokine imbalance, chronic low-grade inflammation, lipotoxicity, and mitochondrial oxidative stress [24,188,214,215]. A related systemic phenotype is metabolic dysfunction-associated steatotic liver disease (MASLD) and its inflammatory form, metabolic dysfunction-associated steatohepatitis (MASH). Longitudinal human data link MASLD with incident CKD [216], while experimental work supports shared insulin resistance, visceral adiposity, RAAS activation, endothelial dysfunction, inflammation, and mitochondrial redox stress. These associations do not prove that hepatic disease itself causes CKD. Likewise, clinical benefits of weight loss, SGLT2 inhibition, or GLP-1RA therapy across metabolic diseases do not establish a direct renal mitochondrial mechanism; rather, they support the translational relevance of a shared cardio–kidney–metabolic–liver axis whose mitochondrial contribution remains to be defined.

Hormonal factors also influence mitochondrial homeostasis. Estrogen has been shown to exert protective effects on mitochondrial function by enhancing antioxidant enzyme activity and regulating mitochondrial calcium handling. These effects may partly explain the lower incidence of CKD observed in premenopausal women [213].

Thyroid hormones represent another important regulator of mitochondrial metabolism. Hypothyroidism, which is common in advanced CKD, is associated with reduced mitochondrial respiration and impaired energy metabolism, potentially contributing to accelerated renal decline [213,217,218,219].

Dietary interventions aimed at improving mitochondrial metabolism—such as caloric restriction, ketogenic diets, and increased intake of antioxidant-rich foods—have been proposed as complementary strategies for slowing CKD progression. Although clinical evidence remains limited, these approaches highlight the importance of metabolic health in maintaining renal mitochondrial function [187,214,220].

## 10. Conclusions and Future Perspectives

Chronic kidney disease is characterized by reciprocal amplification between redox imbalance and mitochondrial dysfunction. ETC injury, NOX- and RAAS-dependent signaling, Na^+^/K^+^-ATPase-linked signaling, impaired mitochondrial quality control, ferroptosis, and metabolic-inflammatory stress converge on ATP deficiency, ROS accumulation, inflammation, and fibrosis. The mechanistic evidence is strongest in animal and cellular models, whereas human validation remains uneven for most redox biomarkers and direct mitochondria-targeted interventions.

The limited clinical literature should be viewed as an essential test of translatability rather than as a separate body of evidence: it shows where biological plausibility has produced early signals and where model-based expectations have not yet yielded durable renal outcomes. Progress will require standardized biomarkers, demonstration of renal mitochondrial target engagement, appropriately powered and longer trials, clinically meaningful kidney endpoints, and experimental models that better reproduce age, comorbidity, background therapy, and established fibrosis. This evidence-aware continuum from mechanism to translation is likely to be more informative than either preclinical or clinical evidence considered in isolation.

## Figures and Tables

**Table 1:** Evidence profile of the literature included in this narrative review. Mixed-design primary studies were classified according to the dominant/highest translational evidence level.

Evidence Category	References (n)	% of Primary Studies	Typical Contribution	Main Limitation
Human clinical/observational/translational	46	37.7%	Clinical associations, biomarkers, intervention outcomes	Heterogeneous populations and assays; mechanistic causality often indirect
Animal/in vivo experimental	60	49.2%	Causal pathway interrogation and therapeutic proof-of-concept	Species-, model-, dose-, and injury-context dependence
In vitro/cellular	16	13.1%	Molecular mechanism and target validation	Limited tissue complexity and uncertain in vivo exposure relevance
Reviews, guidelines, methodological/background	94	Not included in primary-study denominator	Context, synthesis, definitions, and clinical framing	Secondary evidence; does not replace direct validation

**Table 2:** Redox and mitochondrial biomarkers in chronic kidney disease: mechanistic meaning, analytical approaches, translational status, and major limitations.

Biomarker	Biological Matrix	Pathophysiological Domain Reflected	Main Analytical Method	Translational Status/Potential Use	Main Limitations
F2-isoprostanes	Plasma; urine	Global lipid peroxidation and in vivo oxidative stress	GC–MS; LC–MS/MS	Research-grade assessment of systemic oxidative burden; human associations exist, but no validated CKD decision threshold	Limited routine availability; mass spectrometry often required; values may be influenced by pre-analytical variability
15-F_2_t-isoprostane	Urine	Systemic lipid peroxidation	LC–MS/MS; GC–MS	Non-invasive research marker of lipid peroxidation; clinical interpretation remains exploratory	Less widely standardized than routine clinical biomarkers; interpretation may vary across assays
8-OHdG	Urine; plasma	Oxidative DNA damage	ELISA; HPLC; LC–MS/MS	Candidate marker of oxidative DNA injury; associations do not establish kidney-specific causality	Affected by smoking, inflammation, and metabolic comorbidities; assay heterogeneity limits comparability
Advanced oxidation protein products (AOPPs)	Serum; plasma	Protein oxidation and inflammation-associated oxidative damage	Spectrophotometric assay; ELISA-based methods	Research marker of oxidative-inflammatory burden; low renal specificity limits stand-alone use	Lower specificity; influenced by systemic inflammation and albumin oxidation
Circulating mtDNA	Plasma; serum	Mitochondrial injury, cell stress, and DAMP-mediated inflammation	qPCR; digital PCR	Mechanistically attractive marker of mitochondrial injury; clinical risk-stratification role remains unvalidated	Pre-analytical variability; lack of standardized reference ranges; may reflect extra-renal sources
Urinary mtDNA	Urine	Renal mitochondrial injury and tubular stress	qPCR; digital PCR	Potentially kidney-proximal mitochondrial injury marker; promising for mechanistic studies and AKI-to-CKD research, but not standardized for routine CKD care	Urine handling strongly affects results; normalization strategies are not fully standardized

Abbreviations: AOPPs, advanced oxidation protein products; GC–MS, gas chromatography–mass spectrometry; HPLC, high-performance liquid chromatography; LC–MS/MS, liquid chromatography–tandem mass spectrometry; mtDNA, mitochondrial DNA; qPCR, quantitative polymerase chain reaction.

**Table 3:** Therapeutic strategies targeting oxidative stress and mitochondrial dysfunction in chronic kidney disease.

Therapy/ Intervention	Main Target/Pathway	Expected Mitochondrial/Redox Effect	Evidence Level/Translational Status	Main Limitation or Caveat
SGLT2 inhibitors	AMPK/SIRT1 signaling; metabolic reprogramming; autophagy-related pathways	Reduce oxidative stress, improve mitochondrial efficiency, and support mitochondrial quality control	Established CKD outcome evidence; mitochondrial/redox mechanisms supported mainly by experimental studies	Clinical benefit cannot be attributed specifically to mitochondrial effects; mechanisms are pleiotropic
RAAS inhibitors	Angiotensin II signaling; NADPH oxidase activation	Lower Ang II-mediated ROS production and limit fibrosis-associated redox injury	Established CKD therapy; redox effects supported by mechanistic and translational studies	Hemodynamic and antifibrotic effects confound attribution to direct antioxidant mechanisms
Bardoxolone methyl/Nrf2 activation	Nrf2-dependent antioxidant transcriptional response	Enhance antioxidant defenses and redox resilience	Human CKD trials demonstrate pathway activity but inconsistent risk–benefit balance	eGFR changes may not equal structural renoprotection; systemic Nrf2 activation produced fluid/heart-failure safety concerns in susceptible patients
MitoQ	Mitochondria-targeted antioxidant activity within the inner mitochondrial membrane	Directly scavenges mitochondrial ROS and may reduce oxidative damage	Strong preclinical renal rationale; very limited CKD-specific human proof-of-concept evidence	Small samples, short exposure, surrogate/vascular endpoints, uncertain renal target engagement, and no CKD-progression outcome evidence
SS-31 (elamipretide)	Cardiolipin stabilization; electron transport chain function	Improves mitochondrial bioenergetics and reduces ROS generation	Preclinical renal evidence plus a small human renovascular proof-of-concept study; no established efficacy for progressive CKD	Highly selected disease/procedural context, small sample, and uncertain generalizability to heterogeneous CKD
Mitochondrial transplantation	Replacement of damaged mitochondria with functional mitochondria	Restores cellular bioenergetics and may reduce inflammation and oxidative injury	Experimental/early-stage preclinical approach	Delivery, mitochondrial uptake, durability, immunologic safety, manufacturing, and standardization remain unresolved
Nanotechnology-based delivery systems	Targeted delivery of antioxidants or bioactive compounds to renal tissue or mitochondria	Increase local drug concentration while limiting systemic toxicity	Early experimental/proof-of-concept stage	Biodistribution, off-target exposure, scale-up, reproducibility, and limited human renal data
Mineralocorticoid receptor antagonists (MRAs)	Aldosterone/mineralocorticoid receptor signaling; NADPH oxidase activation; NLRP3 and profibrotic pathways	Reduce ROS generation, attenuate mitochondrial oxidative stress and inflammatory signaling, and limit fibrosis-associated redox injury	Established renal and cardiovascular benefit in selected CKD populations; mitochondrial/redox mechanisms are supported mainly by experimental and translational studies	Hyperkalemia and changes in renal function may limit use; clinical benefit cannot be attributed specifically to mitochondrial or antioxidant effects
GLP-1 receptor agonists (GLP-1RAs)	GLP-1 receptor signaling; metabolic reprogramming; weight and glycemic control; anti-inflammatory pathways	Reduce lipotoxicity and inflammatory burden and may improve mitochondrial substrate handling, bioenergetics, and redox homeostasis	Clinical kidney outcome benefit in type 2 diabetes with CKD and broader metabolic disease settings; direct renal mitochondrial evidence remains predominantly experimental	Pleiotropic metabolic and hemodynamic effects make direct mitochondrial target attribution difficult; CKD-specific mitochondrial target engagement has not been established

Abbreviations: AMPK, AMP-activated protein kinase; CKD, chronic kidney disease; eGFR, estimated glomerular filtration rate; GLP-1RA, glucagon-like peptide-1 receptor agonist; MitoQ, mitochondria-targeted coenzyme Q10 derivative; MRA, mineralocorticoid receptor antagonist; Nrf2, nuclear factor erythroid 2–related factor 2; RAAS, renin–angiotensin–aldosterone system; ROS, reactive oxygen species; SGLT2, sodium–glucose cotransporter 2; SIRT1, sirtuin 1; SS-31, Szeto–Schiller 31 peptide (elamipretide).

## Data Availability

No new data were created or analyzed in this study. Data sharing is not applicable to this article.

## Abbreviations

The following abbreviations are used in this manuscript:

8-OHdG	8-hydroxy-2′-deoxyguanosine
ACSL4	Acyl-CoA synthetase long-chain family member 4
ADPKD	Autosomal Dominant Polycystic Kidney Disease
AGEs	Advanced Glycation End products
AhR	Aryl hydrocarbon receptor
AKI	Acute Kidney Injury
AMPK	AMP-activated protein kinase
AOPPs	Advanced Oxidation Protein Products
AP-1	Activator Protein-1
AT_1_R	Angiotensin II Type 1 Receptor
ATP	Adenosine Triphosphate
BNIP3	BCL2 Interacting Protein 3
CFTR	Cystic Fibrosis Transmembrane Conductance Regulator
cGAMP	Cyclic GMP–AMP
cGAS	Cyclic GMP–AMP Synthase
CKD	Chronic Kidney Disease
CTGF	Connective Tissue Growth Factor
DAMPs	Damage-Associated Molecular Patterns
DKD	Diabetic Kidney Disease
DNA	Deoxyribonucleic Acid
DRP1	Dynamin-Related Protein 1
EGCG	Epi-Gallo-Catechin-3-Gallate
eGFR	Estimated Glomerular Filtration Rate
ELISA	Enzyme-Linked Immunosorbent Assay
ER	Endoplasmic Reticulum
ETC	Electron Transport Chain
EZH2	Enhancer of Zeste Homolog 2
F2-IsoPs	F2-isoprostanes
FAO	Fatty Acid Oxidation
FIS1	Fission 1
FSGS	Focal Segmental Glomerulosclerosis
FUNDC1	FUN14 Domain Containing 1
GC–MS	Gas Chromatography–Mass Spectrometry
GFR	Glomerular Filtration Rate
GLP-1RA	glucagon-like peptide-1 receptor agonist
GPX4	Glutathione Peroxidase 4
GSH	Glutathione
H_2_O_2_	Hydrogen Peroxide
H3K27ac	Histone H3 Lysine 27 Acetylation
H3K27me3	Histone H3 Lysine 27 Trimethylation
HIF-1α	Hypoxia-Inducible Factor 1-alpha
HO-1	Heme Oxygenase-1
IP3R	Inositol 1,4,5-trisphosphate Receptor
IS	Indoxyl Sulfate
LC3	Microtubule-Associated Protein Light Chain 3
LC–MS/MS	Liquid Chromatography–Tandem Mass Spectrometry
LIR	LC3-interacting region
LN	Lupus Nephritis
MAMs	Mitochondria-Associated Membranes
MASH	metabolic dysfunction-associated steatohepatitis
MASLD	metabolic dysfunction-associated steatotic liver disease
MBG	marinobufagenin
MCU	Mitochondrial Calcium Uniporter
MFF	Mitochondrial Fission Factor
MFN1	Mitofusin 1
MFN2	Mitofusin 2
miRNAs	MicroRNAs
MitoQ	Mitochondria-targeted Coenzyme Q10 derivative
MN	Membranous Nephropathy
mPTP	Mitochondrial Permeability Transition Pore
MQC	Mitochondrial Quality Control
MR	Mineralocorticoid Receptor
MRA	mineralocorticoid receptor antagonist
mtDNA	Mitochondrial DNA
mTOR	Mammalian Target of Rapamycin
MTP18	Mitochondrial Protein 18
NADPH	Nicotinamide Adenine Dinucleotide Phosphate
NDP52	Nuclear Dot Protein 52
NF-κB	Nuclear Factor-kappa B
NIX	NIP3-like Protein X
NLRP3	NLR Family Pyrin Domain Containing 3
NOX	NADPH Oxidase
NOX2	NADPH oxidase 2
NOX4	NADPH Oxidase 4
NRF1	Nuclear Respiratory Factor 1
Nrf2	Nuclear Factor Erythroid 2–Related Factor 2
NRF2	Nuclear Respiratory Factor 2
O_2_•^−^	Superoxide Radical
OH•	Hydroxyl Radical
OPA1	Optic Atrophy Protein 1
OPTN	Optineurin
OXPHOS	Oxidative Phosphorylation
pCS	p-cresyl Sulfate
p-DRP1S616	Phosphorylated DRP1 at Serine 616
p-DRP1S637	Phosphorylated DRP1 at Serine 637
PGC-1α	Peroxisome Proliferator-Activated Receptor Gamma Coactivator-1 Alpha
PINK1	PTEN-Induced Kinase 1
PKD1	Polycystic Kidney Disease 1
PKD2	Polycystic Kidney Disease 2
PMPCB	Mitochondrial Processing Peptidase Subunit Beta
PPAR-α	Peroxisome Proliferator-Activated Receptor Alpha
PUFAs	Polyunsaturated Fatty Acids
qPCR	Quantitative Polymerase Chain Reaction
RAAS	Renin–Angiotensin–Aldosterone System
RNA	Ribonucleic Acid
ROS	Reactive Oxygen Species
SAM	S-adenosylmethionine
SGLT2	Sodium–Glucose Cotransporter-2
SH3YL1	SH3 Domain Containing YSC84-Like 1
SIRT1	Sirtuin 1
SIRT3	Sirtuin 3
SODs	Superoxide Dismutases
SS-31	Szeto–Schiller 31 peptide (elamipretide)
STING	Stimulator of Interferon Genes
TCA	Tricarboxylic Acid Cycle
TFAM	Mitochondrial Transcription Factor A
TGF-β	Transforming Growth Factor-beta
TLR9	Toll-Like Receptor 9
UUO	unilateral ureteral obstruction
VDAC	Voltage-Dependent Anion Channel
